# Numerical analysis of blood flow through stenosed microvessels using a multi-phase model

**DOI:** 10.1016/j.heliyon.2024.e29843

**Published:** 2024-04-21

**Authors:** Yuhong Zhao, Jue Xie

**Affiliations:** Department of Blood Transfusion, The Frist Affiliated Hospital, Zhejiang University School of Medicine, Hangzhou, 310003, Zhejiang, China

**Keywords:** Blood flow, Stenosis, Microvessel, Wall shear stress, Volume fraction

## Abstract

Blood flow in arterioles have attracted considerable research attention due to their clinical implications. However, the fluid structure interaction between red blood cells and plasma in the blood poses formidable difficulty to the computational efforts. In this contribution, we seek to represent the red blood cells in the blood as a continuous non-Newtonian phase and construct a multi-phase model for the blood flow in microvessels. The methods are presented and validated using a channel with sudden expansion. And the resulting blood flow inside a stenosed microvessel is investigated at different inlet velocity amplitudes and hematocrits. It is show that the increase of both inlet velocity amplitude and inlet hematocrit leads to longer and thicker cell-rich layer downstream the stenosis. Besides, it is found that the maximum values of wall shear stress scales up with inlet velocity amplitudes and hematocrits. These results show the validity of the proposed computational model and provide helpful insights into blood flow behaviors inside stenosed vessels.

## 1 Introduction

Atherosclerosis, or the deposit of fatty materials in the inner layers of arteries as a result of hyperlipidemia and lipid oxidation, has been a key health concern in many countries [1]. The formed plaque consists of fatty material in the core and fibrous material in the cap, and leads to the sudden narrowing of blood vessels, or vascular stenosis, and the hardening of artery walls [2]. Rupture of the fibrous cap, or plaque rupture exposes the highly thrombogenic fatty core to the blood and leads to the formation of thrombosis and possible heart attacks and strokes [3]. It has been established that [4] plaque rupture usually occurs at the cap surface of the plaque, where the cap is thinnest and mostly infiltrated by foam cells, namely cap shoulders [5], [6].

Though the underlying mechanisms are not fully understood, plaque rupture is generally said to result from the combined action of intrinsic plaque features determining its vulnerability to rupture, and extrinsic imposed stresses as rupture triggers [7]. Indeed, elevated wall shear stress in partially occluded vessels is shown to be closely related to the adhesion and aggregation of platelets [8] and thus has been considered a major contributor to artery thrombosis [9], [10]. To this end, blood flow in various arteries has been extensively covered in the last decades [11], [12], [8].

In recent years, however, increasing scientific attention is paid to the blood flow in arterioles whose inner diameters are up to a few hundred microns [13], [14], [15], [16], [17]. In this situation, several concerns are raised. Firstly, blood is a kind of complex fluid mainly composed of plasma, red blood cells (RBCs), white blood cells, and platelets. In microvessels whose diameters are comparable to the characteristic sizes of RBCs, blood flow is pronouncedly non-Newtonian, [18] showing interesting flow phenomena including the Fahraeus effect [19], [20], the Fahraeus–Lindqvist effect [21], [22], shear-thinning rheology [23], [24], viscoelasticity [25], [26], viscoplasticity [27], [28], and thixotropy [29], [30], [31]. Secondly, since plasma and RBCs are of dominant volume fractions in whole blood, theoretically and computationally blood are usually considered as a suspension of discrete RBCs into continuous plasma. In this spirit, various fluid-structure interaction methods are then used to investigate cell dynamics through stenosed microvessels, including the immersed boundary method, the dissipative particle dynamics method, and the smoothed particle hydrodynamics method [15], [32], [33], [34]. However, since the diameters of microvessels are still large compared to the sizes of RBCs, a large number of RBCs are usually involved in the considered blood flow. Hence, the calculation and tracking of the dynamic behaviors of each RBC would be extremely resource-demanding and practically impossible.

To make a compromise, it is helpful to consider the blood as a two-fluid system. The plasma is considered the primary continuous phase with Newtonian behaviors, while RBCs are approximated using a second continuous phase with complex non-Newtonian properties [35]. With this method, blood flow is readily amenable to computational fluid dynamics with appropriate determination of phase rheologies and interactions [36]. Here in this contribution, we would like to investigate blood flow in stenosed microvessels is investigated with the help of this two-fluid approach. The rest of the current contribution is organized as follows. We firstly give a brief review of the two-fluid model. Rheological model of the RBC phase is established and the phase interactions are modeled. The computational model is then validated using a benchmark problem involving blood flow through a channel with sudden expansion. Next, blood flow in stenosed microvessels are investigated with emphasis on the influences inlet velocity amplitude and hematocrit. Discussions are made about the models adopted and the results obtained. Finally, the conclusions are drawn.

## 2 Two-fluid model of blood flow

The two-fluid model of blood is based on the famous mixture theory of multiple continua [37], [38], in which the governing equations of a single continuum phase are generalized to incorporating any number of superimposed continua [39], [40]. The theory has been extensively utilized in the study of various biomechanical problems [41], [42], [43], [44], [45]. With the mixture theory in mind, blood is represented by a two-fluid system: the plasma phase and the RBC phase. And the conservation of mass for the plasma and RBCs can be described [39], [40] in equation (1)

(1) $$\frac{\partial }{\partial t}\left[(1-\phi )\rho_{p}\right]+\nabla \cdot \left[(1-\phi )\rho_{p}v_{p}\right]=0,$$

and equation (2)

(2) $$\frac{\partial }{\partial t}\left(\phi \rho_{r}\right)+\nabla \cdot \left(\phi \rho_{r}v_{r}\right)=0,$$

respectively. In these equations, *ϕ* is the volume fraction of RBCs (hematocrit), and $v_{r}$ and $v_{p}$ are the velocity field for the primary phase (plasma) and the secondary phase (RBCs), respectively. $\rho_{p}$ and $\rho_{r}$ are the densities of pure plasma and pure RBCs, respectively. Similarly, the linear momentum equations for both phases take the form [36], [46]

(3) $$(1-\phi )\rho_{p}\left[\frac{\partial v_{p}}{\partial t}+\left(v_{p}\cdot \nabla \right)v_{p}\right]=\nabla \cdot T_{p}+(1-\phi )\rho_{p}b_{p}+f_{I},$$

and

(4) $$\phi \rho_{r}\left[\frac{\partial v_{r}}{\partial t}+\left(v_{r}\cdot \nabla \right)v_{r}\right]=\nabla \cdot T_{r}+\phi \rho_{r}b_{r}-f_{I},$$

respectively. Equation (3) represents the momentum balance for the plasma phase and equation (4) represents the momentum balance for the RBC phase. Here $T_{p}$ and $T_{r}$ are the Cauchy stress tensor for the plasma phase and the RBC phase respectively, $b_{p}$ and $b_{r}$ are the body forces for the two phases respectively, and $f_{I}$ is the interaction forces between the two phases.

### 2.1 Constitutive relations

As described above, in the context of mixture theory, the constitutive relations for blood are determined by the two component phases: the plasma and the RBCs. Though it has been found that plasma can be viscoelastic under certain flow conditions [47], [48], study also indicates that the viscosity of plasma experiences no significant deviation from a constant value under a variety of shear rates [49]. Hence in the current contribution, the plasma is simply assumed to be Newtonian and its constitutive relation is formulated as [46]

$$T_{p}=\left[-p(1-\phi )+\lambda_{p}(1-\phi )tr\left(D_{p}\right)\right]I+2\mu_{p}(1-\phi )D_{p},$$

where *p* is the hydrodynamic pressure of blood, **I** is the identity tensor, $\lambda_{p}$ and $\mu_{p}$ are the (constant) first and second coefficients of viscosity of plasma, respectively, “tr” stands for the trace of a matrix, and $D_{p}$ is the symmetric part of the velocity gradient of the plasma

$$D_{p}=\frac{1}{2}\left[(\nabla v_{p})+{\left(\nabla v_{p}\right)}^{T}\right],$$

in which the superscript *T* means matrix transpose. Note that due to the general incomprehensibility assumptions for fluid dynamics, it is generally set that

$$tr\left(D_{p}\right)=0.$$

In this situation, it is seen that the viscosity coefficients $\lambda_{p}$ are of no presence in later simulations and the constitutive relation for the plasma can be expressed as

$$T_{p}=-p(1-\phi )I+2\mu_{p}(1-\phi )D_{p}$$

without losing any information.

When it comes to the RBC phase, complicated dynamic behaviors of RBCs as flexible granular materials originated from its microstructures are firstly to be considered. A healthy human RBC assumes a biconcave shape with a diameter of around 7.8μm [50]. Enclosing the cytoplasma with a viscosity several times larger than that of the plasma in general, membrane of the RBC involves a lipid bilayer and the attached cytoskeleton [51]. Due to the diffusion of lipids and proteins within the lipid bilayer [52] and the absence of organelles and filaments inside the cytoplasma [51], RBC membrane manages to resist bending moments but fails to sustain in-plane static shear stress [26], [53], [54]. Thus, dynamic behaviors of RBCs inside blood are then affected by membrane rheology, and fluid flow inside and outside the membrane [50]. Yet, apart from undergoing deformations, translations, and rotations as isolated particles, RBCs interact with each other in blood flow, resulting in the formation and disintegration of rouleaux [55] and/or more complex networks [56], [57], [58], rendering extra difficulties to the dynamic description of RBCs [59]. According to the mixture theory for multi-component systems, the RBCs in blood flow can be represented as an anisotropic non-linear density-gradient-type fluid [60], which originates from the theory of interacting continua for granular materials [61], [62]. The corresponding stress tensor for the RBC phase is [63], [46]

$$T_{r}=\left[-p\phi +\lambda_{r}\phi tr\left(D_{r}\right)\right]I+2\mu_{r}\phi D_{r},$$

in which the $\mu_{r}$ and $D_{r}$ are expressed using [60]

$$\mu_{r}=\frac{1}{2}\left[\mu_{\infty }+(\mu_{0}-\mu_{\infty })\frac{1+\ln(1+k\overset{˙}{\gamma })}{1+k\overset{˙}{\gamma }}\right](1+\phi )$$

and

$$\overset{˙}{\gamma }=\sqrt{2\;tr\left(D_{r}^{2}\right)}$$

respectively. In the above equations, $D_{r}$ is the symmetric part of the velocity gradient of RBCs, and $\overset{˙}{\gamma }$ is the generalized shear rate. The values of $\mu_{0}$, $\mu_{\infty }$ and *k* are thought as functions of the volume fractions of the RBC phase *ϕ* [6]. According to polynomial fitting to the experimental results by Brooks et al. [64], [46], the material property coefficients $\mu_{0}$ and $\mu_{\infty }$ are represented by

$$\left\{ \begin{array}{cc} \mu_{0} & =537.002\;\phi^{2}+55.006\;\phi -0.129,\\ \mu_{\infty } & =27.873\;\phi^{2}-21.218\;\phi +14.439,\\ k & =11, \end{array}\right.$$

in which *k* is determined through the theoretical model by Yeleswarapu et al. [65].

### 2.2 Phase interactions

The phase interactions are generally formulated based on the hydrodynamic forces experienced by a rigid particle in a surrounding fluid flow due to the gradients of the density, pressure, and velocity of the fluid flow, the differences of density, spin, velocity and acceleration between the particle and the local fluid flow, and the motion history of the particle and the fluid flow, and etc. [66]. In the current contribution, however, the drag force, the Saffman's shear-lift force [67], and the spin-lift force [68] are considered for the phase interactions. Hence, the phase interaction forces are [69], [36]

(5) $$f_{I}=\frac{9\mu_{p}}{2a^{2}}f(\phi )\left(v_{r}-v_{p}\right)+\frac{3(6.46){\left(\rho_{p}\mu_{p}\right)}^{1/2}}{4\pi a}\phi {\left[2tr\left(D_{p}^{2}\right)\right]}^{-1/4}D_{p}\left(v_{r}-v_{p}\right)+\frac{3}{4}\rho_{p}\phi \left(W_{r}-W_{p}\right)\left(v_{r}-v_{p}\right),$$

where a=8μm is the diameter of RBCs, f(ϕ)=ϕ(1+6.55ϕ) is the hindrance function determined from the polynomial fitting correlations from experimental and theoretical results [70], [71], [72], and $W_{p}$ and $W_{r}$ refer to the spin tensors of the plasma phase and the RBC phase respectively [73], [68].

Some notes are to be made here. Firstly, though various forces are involved in the interaction, only three of them are considered here. The drag force are considered due to the fact that the blood flow velocity in microvessels are small, indicating an important role viscous drag. The two lift forces are taken into consideration to account for the non-homogeneous distribution of the RBC phase inside the microvessels towards the core [74], [75], the cell free layer near vessel walls [76], [77], [78], and the related local accumulation of platelets and other components in the blood [79]. Secondly, various other forces can be added readily to the phase interactions in equation (5), including the surface tension, the virtual mass, the Basset forces related to the flow acceleration, to name just a few. Last but not the least important, considering the phase interactions influenced by the volume fractions of the RBC phase *ϕ*, corrections are generally introduced to the theoretical expressions of the involved terms. However, those corrections are inherently related to the multiscale microstructure and anisotropic macroscopic motion of RBCs, the complex flow conditions in practical microvessels, and the nonlinear nature of the blood flows. Though some corrections have already been obtained by extracting theoretical fitting of experimental results, there are still corrections to be elucidated [60]. Besides, consistency between the different versions of corrections in terms of the same sort of contribution to the phase interaction has not been fully checked. And the physical mechanisms and implications underlying those corrections are yet to be revealed.

## 3 Numerical simulations

The current contribution is meant for the computational investigation of blood flow dynamics and RBC distribution inside stenosed microvessels as a result of plaque formation or angiomas. Adopting the two-fluid multi-phase model described above, the computational analysis is conducted with the help of Fluent (ANSYS, Inc., Canonsburg, PA). The meshes utilized are created by ICEM-CFD (ANSYS, Inc., Canonsburg, PA) for the geometries considered. The plasma is set as the primary phase and the RBCs are chosen as the secondary phase. As for material properties, a density of $1027\;kg/m^{3}$ and a viscosity of 0.96cP are adopted for the plasma and the density of RBCs is set to be $1093\;kg/m^{3}$ [46]. The viscosity of RBCs and the interaction forces are implemented through user-defined functions in Fluent.

### 3.1 Validation for microvessels with sudden expansion

**Figure 1:**
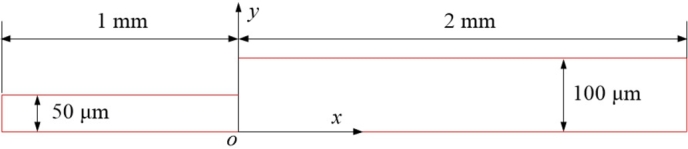
Geometry of the microvessel with sudden expansion.

To validate the established computational model, a benchmark case [80], [46] involving the blood flow in a rectangular microvessel with sudden expansion is examined. Geometry of the microvessel is shown in Fig. 1. The part of the microvessel upstream the expansion has a height of 100μm and a length of 1mm, while the downstream part of the microvessel has a height of 200μm and a length of 2mm. Note that in Fig. 1, only half of the microvessel geometry is shown due to the assumption of flow symmetry. A uniform velocity distribution with an amplitude of Vt=0.833m/s and a uniform hematocrit of Ht=20% are set at the inlet (leftmost boundary in the figure) of the microvessel and a zero pressure condition is maintained at the outlet (leftmost boundary in the figure). The no-slip condition is set at the upper walls of the microvessel, and symmetric conditions are imposed at the symmetry line. A coordinate system *xoy* is established at the point of expansion with the *ox* axis directing downstream along the symmetry line and the *oy* axis directing towards the vessel wall.

**Figure 2:**
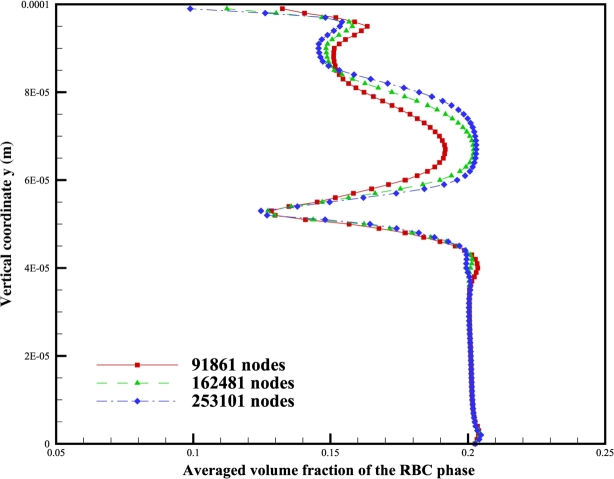
Volume fraction profile of the RBC phase in the *y* direction at the cross-section *x* = 0.02 *mm*.

**Figure 3:**
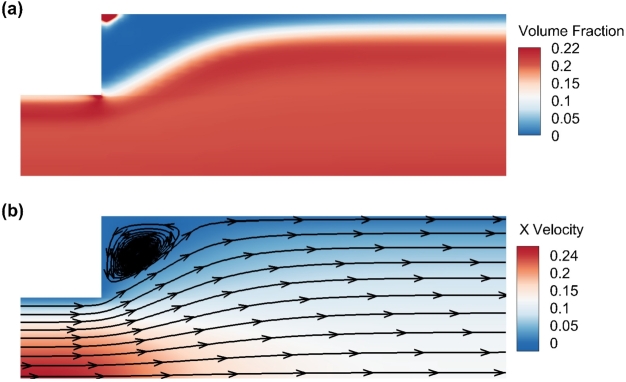
Volume fraction of the RBCs (a) and streamline of the plasma velocity (b) according to simulation. The scale bars represent the volume fraction and the *x*-velocity component respectively.

Adopting different mesh sizes with different number of nodes, averaged volume fraction profile of the RBC phase along *y* direction, at the cross section of x=0.02mm just downstream the expansion point, is shown in Fig. 2. Quick visual comparison of the profiles indicates a convergence between 162,481 nodes and 253,101 nodes. Therefore, 162,481 nodes are used in later simulations to ensure mesh independence. With these settings, resulting distributions of the volume fractions of RBCs and the streamline of plasma velocity are shown in Fig. 3. A cell-free layer adjacent to the vessel wall is developed downstream of the expansion, as shown in the distribution of volume fraction of RBCs shown in Fig. 3a). This is consistent with the experimental observations by Zhao et al. [80]. A recirculation zone at the corner of the expansion is also developed. Only a small area of RBC accumulation at the corner is shown by the simulation results in Fig. 3b). This again agrees with the observations by Zhao et al. [80] that platelet-sized particles are locally accumulated at the corner. These findings validate our models and simulations. Note that here to include the effect of gravity, which is obvious in the experimental results of Zhao et al. [80] gravity terms are added to the equations (3) and (4) as part of the body forces $b_{p}$ and $b_{r}$, respectively.

### 3.2 Blood flow in stenosed microvessels

**Figure 4:**
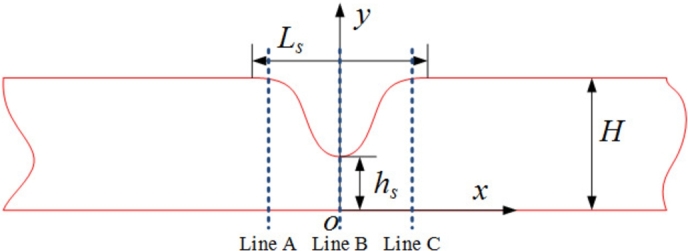
Geometry of the stenosed microvessel considered in the simulation.

With the established and validated model, blood flow in stenosed microvessels is investigated. Geometry of the stenosed microvessel is shown in Fig. 4. Considering practical blood flow in microvessels as axisymmetric, only half of the stenosed microvessel is shown. A coordinate system *xoy* is created with the origin *o* located at the point of maximum stenosis. The axis *ox* is directed downstream along the axisymmetry line of the vessel, and the axis *oy* is directed towards the vessel wall. The vessel profile is then described by

$$y(x)=\frac{H+h_{s}}{2}-\frac{\left(H-h_{s}\right)}{2}\cos\left(\frac{2\pi x}{L_{s}}\right),$$

where y(x) is the *y* coordinate of the vessel wall at given axial position *x*, $L_{s}$ is the length of the stenosed region (in the *x*-direction), $h_{s}$ is half the minimum radius of the stenosed region (in the *y*-direction), and *H* is half the vessel radius far from the stenosis.

The upstream and downstream lengths of the stenosis are set long enough (about 20*H*) to ensure fully developed flow in the vessel at the inlet and the outlet. Two dimensionless parameters are used to describe the stenosis region: the stenosed-height ratio $\eta_{h}=h_{s}/H$, and the stenosed-length ratio $\eta_{L}=L_{s}/H$. In the current contribution, these quantities are chosen to be H=50μm, $h_{s}=25\;\mu \text{m}$, $L_{s}=70\;\mu \text{m}$. As a result, we have $\eta_{h}=0.5$ and $\eta_{L}=1.4$. Both the upstream and downstream length of the microvessel from the point of minimum lumen are 500μm.

**Figure 5:**
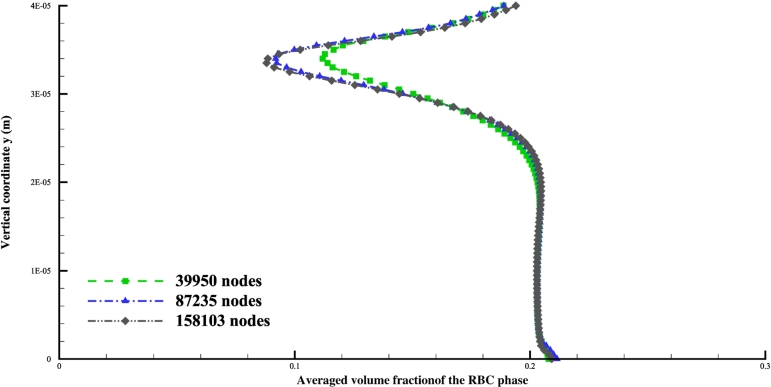
Volume fraction profile of the RBC phase in the *y* direction at the cross-section x=25μm.

Setting the inlet velocity amplitude Vt=0.2m/s and the inlet volume fraction of the RBC phase Ht=20%, and changing meshing sizes, we obtain the distribution of the volume fraction of the RBC phase along the cross section of coordinate x=20μm, and plot the results in Fig. 5. It is seen that the mesh with 158,103 nodes manages to produce accurate results and is thus been exclusively utilized in later simulations.

The subsequent simulations of the blood flow in stenosed microvessels are based on the validated computational model described and validated above. To make it convenient for the characterization of simulation results, three cross-sections along the vessel axis are chosen, which are shown in Fig. 4 by line *A*, line *B*, and line *C*, respectively. Note that in Fig. 4, *x* is the axial coordinate with the origin located at *O*, and *y* is the radial coordinate. Line *B* is marking the narrowest point at the stenosis expressed by x=0. Line *A* is located at x=−30μm, while line *C* is located at x=30μm.

In the simulation, a uniform velocity profile with vanishing radial component is applied at the inlet. The applied velocity amplitude is set to be *Vt*. Zero pressure condition is imposed at the outlet of the microvessel. No-slip condition is enforced at the vessel wall. A uniform hematocrit of amplitude *Ht* is adopted at the inlet.

**Figure 6:**
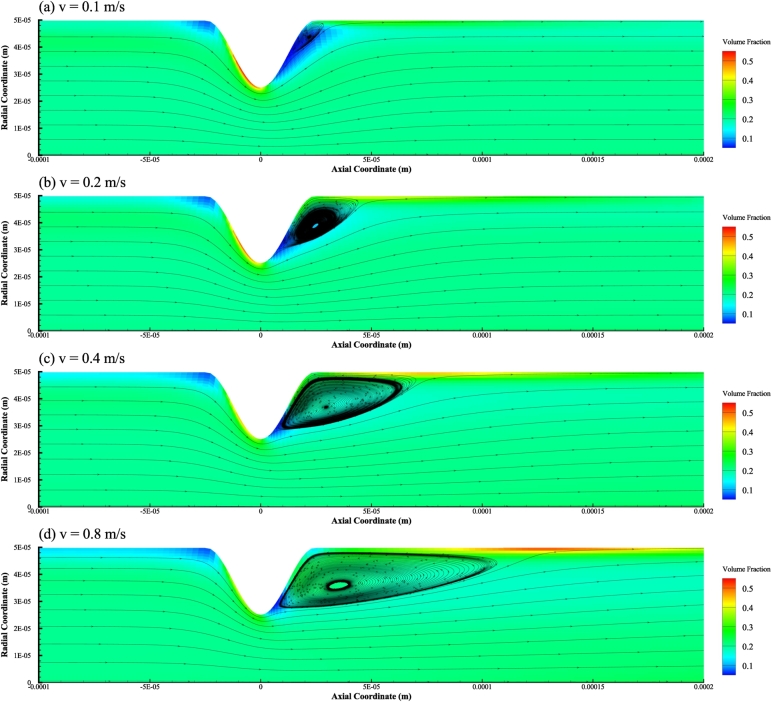
Volume fraction of the RBC phase and streamline of the plasma phase for different inlet velocity amplitude *Vt* and identical inlet hematocrit of *Ht* = 20%.

In the first place, volume fraction of the RBC phase in the blood flow is investigated. With the inlet hematocrit set to be Ht=20%, and the inlet velocity amplitude set to be Vt=0.1m/s, Vt=0.2m/s, Vt=0.4m/s, and Vt=0.8m/s, the resulting volume fractions of the RBC phase inside the stenosed microvessel are calculated and plotted in Fig. 6 a), b), c), and d), respectively. The corresponding velocity streamline patterns of the plasma phase in the considered flow are superimposed onto the plot as well.

It is clear that a cell-free region, i.e., the region with low volume fraction of the RBC phase, develops downstream the stenosis, which is accompanied by the presence of a recirculation region for the plasma phase. With the increase of inlet velocity amplitude *Vt*, the downstream cell-free region of the RBC phase becomes increasingly concentrated on the downstream part of the stenosis. At the same time, axial length of the recirculation region of the plasma phase increases. On the other hand, it is observed that a cell-rich region along the vessel wall with a finite axial length and a small radial thickness is developed. With the increase of inlet velocity amplitude *Vt*, axial length of the cell-rich region increase and the starting point of the cell-rich region moves downstream to some degree. It should be noted that the recirculation region of the plasma phase is closely related to the cell-rich region of the RBC phase. Inside the recirculation region of the plasma phase, volume fraction of the RBC phase is relatively low. And immediately beyond the recirculation region, RBCs tends to accumulate in a narrow region along the straight vessel wall. Beyond the cell-rich region, it is found that volume fraction of the RBC phase returns to the normal state in the sense that influence of the stenosis upon the distribution of RBC inside the microvessel is negligible. Besides, it can be seen that a small cell-free region is present at the immediate upstream of the stenosis, and this region is not much affected by different amplitudes of inlet velocity. An interesting point yet to note is that along the upstream part of the stenosis, a cell-rich region is developed. Along with the increase of inlet velocity amplitude *Vt*, thickness of this upstream cell-rich region decreases. In the case that Vt=0.8m/s, the cell-rich region is nearly invisible.

**Figure 7:**
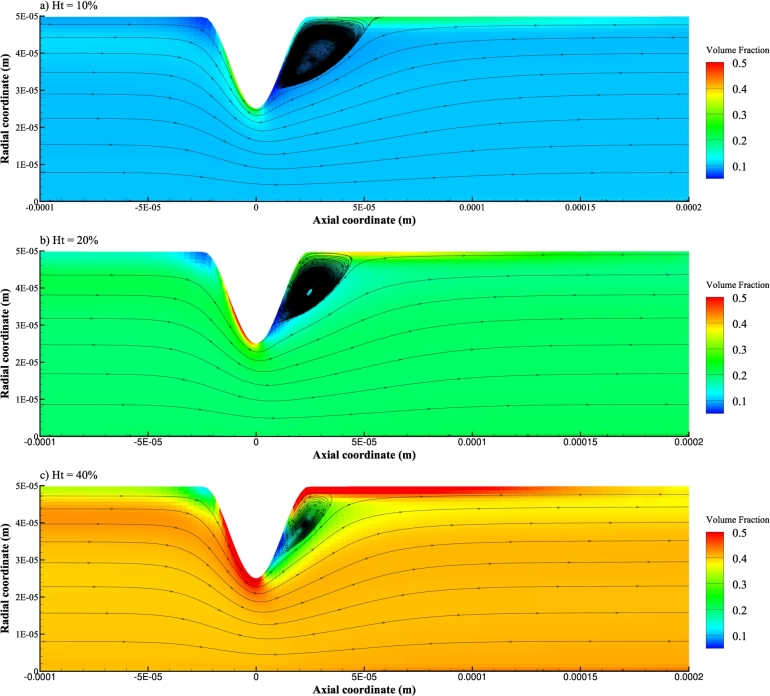
Volume fraction of the RBC phase and streamline of the plasma phase for different inlet hematocrit *Ht* and identical inlet velocity amplitude of *Vt* = 0.2 *m*/*s*.

In a different situation, the inlet velocity amplitude is fixed to be Vt=0.2m/s, and the inlet hematocrit assumes different values of Ht=10%, Ht=20%, and Ht=40%. The resulting contour of the volume fraction of the RBC phase and the velocity streamline of the plasma phase are shown in Fig. 7 a), b), and c), respectively. With the increase of inlet hematocrit *Ht*, it is obvious that the recirculation zone of the plasma phase tends to contract and so does the cell-free region of the RBC phase. At the same time, the cell-rich region on the upstream part of the stenosis expands, indicating that more RBCs accumulate in the relevant region. Moreover, cell-rich region downstream the stenosis expands significantly.

**Figure 8:**
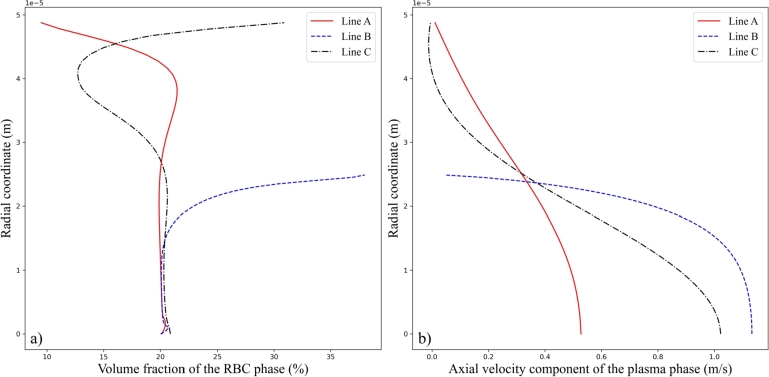
Volume fraction of the RBC phase (a) and axial velocity component of the plasma phase (b) at the three sections *A*, *B*, and *C* under the inlet hematocrit *Ht* of 20% and the inlet velocity amplitude *Vt* of 0.2 *m*/*s*.

In detail, volume fraction of the RBC phase and velocity of the plasma phase across the three defined cross-sections *A*, *B*, and *C* are shown in Fig. 8 a) and b), respectively. The adopted inlet hematocrit is Ht=20%, and the inlet velocity amplitude is Vt=0.2m/s. At cross-section *A* slightly before the stenosis, the volume fraction of the RBC phase starts from a relatively low value near the vessel wall, rapidly increases to its maximum in an intermediate radial position, and then gradually decreases to the central value at the vessel axis. The velocity of the plasma phase increases monotonically to its maximum in the radial direction. As for the cross-section *B* at the stenosis, steep increase of the velocity of the plasma phase is observed, while volume fraction of the RBC phase exhibits a steep decrease. This corresponds to the accumulation of RBCs along the vessel wall around the stenosis. When it comes to the cross-section *C* downstream the stenosis, RBC accumulation at vessel wall is observed, while the presence of recirculation in the plasma phase is exemplified by the local decrease of velocity along Line *C*.

**Figure 9:**
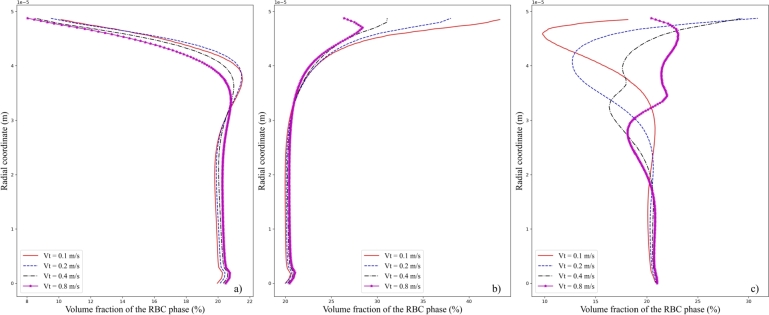
Volume fraction of the RBC phase at the three cross-sections a) *A*, b) *B*, and c) *C* under the inlet hematocrit *Ht* of 20% at different inlet velocity amplitude *Vt*.

Secondly, with the value of *Ht* at the inlet to be 20%, different values of the inlet velocity amplitude *Vt* are adopted, and the resulting distribution of volume fraction of the RBC phase across the cross-sections *A*, *B*, and *C* are shown in Fig. 9, respectively. As for the cross-section *A*, as shown in Fig. 9a), the distribution of volume fraction of the RBC phase is not much affected as *Vt* is changed. With the increase of *Vt*, a slight decrease of the volume fraction of the RBC phase near the vessel wall is observed, while a slight increase in its volume fraction is shown at the vessel axis. This is actually consistent with the law of mass conservation for the blood flow in the microvessel. At the cross-section *B*, as shown in Fig. 9b), the distribution of volume fraction of the RBC phase near the vessel wall is affected slightly. This is obvious for the case of Vt=0.8m/s. A local bump is in the profile of volume of the RBC phase. In contrast, for the cases of Vt=0.1m/s,0.2m/s, and 0.4m/s, no local bumps are seen. When it comes to the cross-section *C*, as shown in Fig. 9c), the distribution profile of volume fraction of the RBC phase across the cross-section changes a lot. Though the value of volumes fraction of the RBC phase at the vessel axis are approximate the same for different values of *Vt*. With low values of inlet velocity amplitude, say Vt=0.1m/s, a bump in the curve is seen. As a result, a local minimum of volume fraction of the RBC phase is present near the vessel wall. Beyond the bump region, the change of volume fraction of the RBC phase is very small. We can then call this region the core region in the microvessel. When *Vt* increases, say Vt=0.4m/s, multiple bumps appear in near the vessel wall before the volume fraction of the RBC phase approaches the core region. It should be noted that, the radial extent of the core region is not much affected by the different values of *Vt*. This can be partly attributed to the gradually contracting recirculation region of the plasma phase, as shown in Fig. 7. Indeed, outside the recirculation region, the velocity profile is not much affected by the presence of the stenosis.

**Figure 10:**
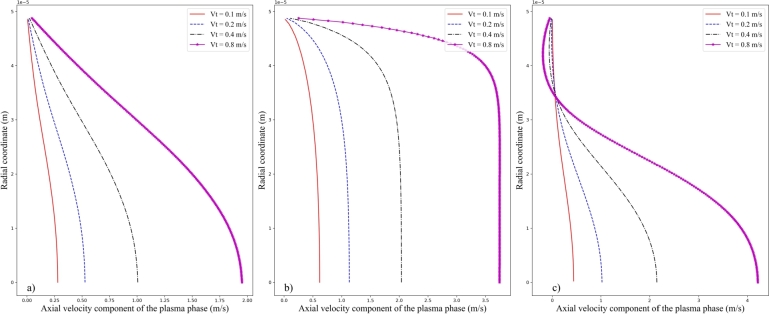
Axial velocity component of the plasma phase at the three cross-sections a) *A*, b) *B*, and c) *C* under the inlet hematocrit *Ht* of 20% at different inlet velocity amplitude *Vt*.

Further information can be extracted by looking at the velocity profile across the three cross-sections *A*, *B*, and *C* at different values of *Vt* and fixed value of Ht=20%, as shown in Fig. 10 a), b), and c), respectively. At the cross-section *A*, a monotonic radial increase of the axial velocity component from zero values at vessel wall to its maximum at the vessel axis is observed. The overall trend of radial variation is similar at different values of *Vt*. For cross-section *B* at the stenosis, a nearly flat profile of the axial velocity component is seen at the core region of the microvessel. Near the vessel wall, a steep increase of the axial velocity component from zero to the plateau value is observed as a result of the narrowing of flow passages. At cross-section *C*, however, the profile of axial velocity component of the plasma phase is obviously affected. With high inlet velocity amplitude, say Vt=0.8m/s, due to the presence of recirculation region shown in Fig. 7, a local decreasing bump of the velocity profile is seen. And outside the recirculation region, the axial velocity component recovers its increasing trend to the maximal value at the axis. While for lower values of *Vt*, say Vt=0.1m/s, this local bump is invisible and a monotonic increase of the velocity profile is obtained.

**Figure 11:**
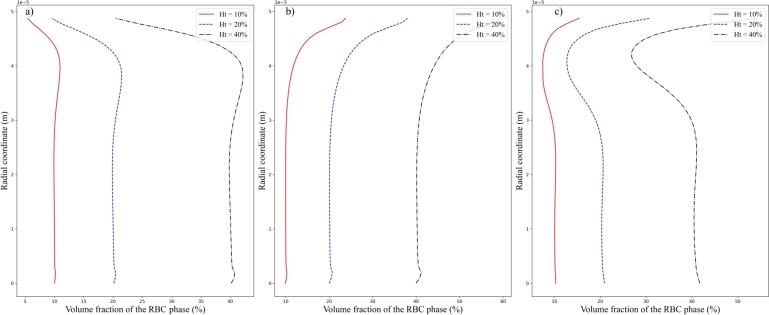
Comparison of the volume fraction of the RBC phase at different location: (a) Line A, (b) Line B, and (c) Line C for different inlet hematocrit of *Ht* = 10%, 20 %, and 40 %, respectively. The inlet velocity amplitudes are set to be identically *Vt* = 0.2 *m*/*s*.

**Figure 12:**
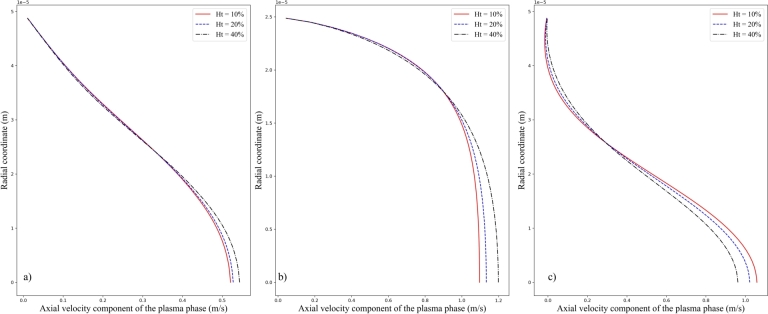
Comparison of the axial velocity component of the plasma phase at different locations: (a) Line *A*, (b) Line *B*, and (c) Line *C* for different inlet hematocrit of *Ht* = 10%, 20 %, and 40 %, respectively. The inlet velocity amplitudes are set to be identically *Vt* = 0.2 *m*/*s*.

Besides, by fixing the inlet velocity amplitude Vt=0.2m/s, influences of the inlet hematocrit *Ht* upon the volume fraction of the RBC phase and axial velocity component of the plasma phase are explored and the results are plotted in Figure 11, Figure 12 respectively. It is inferred from Fig. 11 a), b), and c) that profile of the volume fraction of the RBC phase is not much affected by the increase of inlet hematocrit while the amplitude of the profile is increased. At the same time, it should be noted from Fig. 12 a), b), and c) that the velocity profile is not much affected by the change of inlet hematocrit *Ht*. These results combined with above obtained information indicate that the presence of the RBC phase would not much affect the flow behaviors of the plasma phase. On the other hand, the spatial distribution of the RBC phase is much affected by the flow behavior of the plasma phase.

Finally, wall shear stress due to the flow of the plasma phase is investigated. Indeed, the wall shear stress, $\tau_{wss}$, is often chosen as a quantitative biomarker in the analysis of blood vessels. It is generally defined as the magnitude of the tangential component of the surface traction [81] at vessel wall.

**Figure 13:**
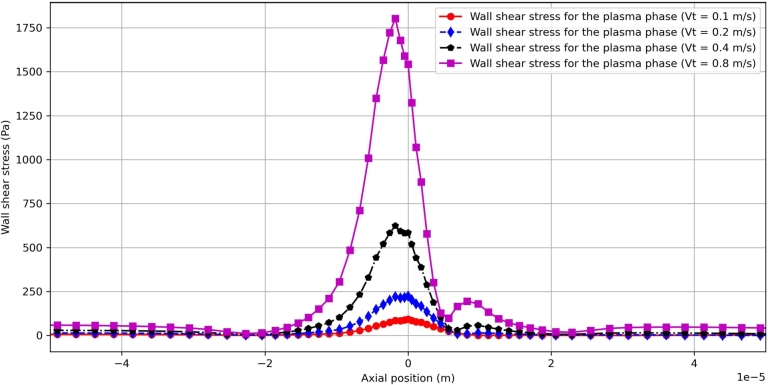
Comparison of the wall shear stress for different inlet velocity amplitude at *Ht* = 20%.

With a fixed inlet hematocrit Ht=20% and different values of inlet velocity amplitude *Vt*, the resulting wall shear stress along the vessel wall is shown in Fig. 13. A clear surge in $\tau_{wss}$ is shown in the axial direction for different values of *Vt*. It should be noted that the maximal value of $\tau_{wss}$ occurs before the narrowest part of the microvessel is attained. This is partly consistent with the upstream accumulation of the RBC phase shown in Fig. 6 and Fig. 7. Interestingly, with the increase of *Vt*, the axial position at which $\tau_{wss}$ peaks tends to move upstream the stenosis. The maximum value of $\tau_{wss}$ increases along with *Vt*. For larger values of *Vt*, say Vt=0.8m/s, a second peak occurs in the downstream part of the stenosis, which is actually related to the presence of recirculation of the plasma phase in this region. Far downstream the stenosis, it is seen that the wall shear stress $\tau_{wss}$ recovers to the undisturbed state.

**Figure 14:**
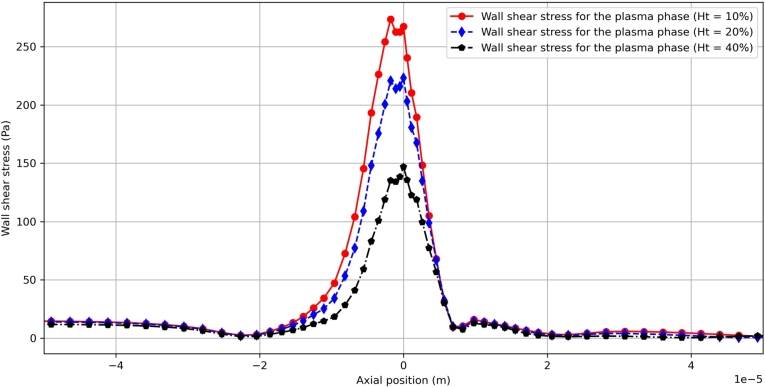
Comparison of the wall shear stress for different inlet hematocrits at *Vt* = 0.2 *m*/*s*.

In a different way, the inlet velocity amplitude *Vt* is held constant Vt=0.2m/s, and the inlet hematocrit *H* is changing from 10% to 40%. The resulting wall shear stress $\tau_{wss}$ is shown in Fig. 14. Though the overall profile is not affected by the applied inlet hematocrit *Ht*, the maximum $\tau_{wss}$ is decreasing with the increase of *Ht*. An interesting point to be noted is that just before the maximum point of stenosis, the wall shear stress experiences a local decrease. Compared the results in Fig. 12 with those in Fig. 5, this seemingly strange local decrease of wall shear stress can be attributed to the formed local recirculation zone, where the RBC phase accumulates and tends to change the local flow behaviors. Similarly, due to the downstream recirculation zone after the stenosis, a local bump in wall shear stress develops just after the stenosis. Besides, it is interesting to note that just before the top of the stenosis, a local drop of wall shear stress can be observed. Again, compared the results in Fig. 12 with those in Fig. 5, this can be explained by the local concentration of the RBC phase. These observations reflect the influence of the RBC phase upon the flow behaviors of the plasma phase, especially near the wall. Yet the detailed interaction between the RBC phase and the plasma phase is to be investigated carefully with the future help of numerical simulations, experimental observations and theoretical analyses.

## 4 Discussions

The two-fluid multi-phase model is adopted here to investigate the blood flow in stenosed microvessels. Though results show that the model is effective in implementation, credible in results, and amenable to further improvements, there are points to be noted and discussed.

Firstly, it must be borne in mind that though the two-fluid model has proven powerful and efficient, it has its own drawbacks. The continuum description of the RBCs is actually a kind of approximation [35], which makes it impossible to capture the detailed dynamic behaviors of red blood cells. Besides, this underlying modeling philosophy has made it hard to incorporating turbulence models in more complex flow conditions, especially when the pulsatile nature of blood flow is considered.

Secondly, vessel geometry is actually crucial for the flow behaviors of blood. Here in the current contribution, however, only idealized stenosis is modeled. This is mainly due to the major goal of the current contribution to computationally analyze the blood flow in stenosed microvessels using the two-fluid mixture model. Indeed, vessel geometries can be highly complex, due to the presence of branches, curvatures, asymmetries, and time-dependent variance [82]. Multiple length scales and time scales are usually needed to completely describe the behaviors of blood vessels, which are not affordable in the current contribution [83]. Besides, it is indeed difficult to obtain accurate vessel geometries in a given physiological conditions. Approximations are then universally adopted. In the computational analysis of blood flows, different idealized vessel geometries are frequently adopted [84], [85], [86].

Thirdly, the rheological models and the phase interactions in the two-fluid description are to be paid special attention. The plasma phase is usually seen as a Newtonian fluid, though recent research has indicated that a viscoelastic model is needed [47], [48]. In addition, already discussed in previous sections, rheological model of the RBC phase is somehow hindered by the complex physics involved. Recent advances in the thixotropic elasto-viscoplastic model [87], [57], [58] has provided insightful clues to the description of real blood behaviors. Besides, it is actually embarrassing that blood should be considered as a whole in any sort of investigations, as various components in the blood actually interact with each other significantly. Proper understanding and incorporation of these interactions can be crucial in the rheological modeling of blood. Last but not the least important, it is to be pointed out that in the two-fluid model, rheological models of the RBC phase are generally semi-empirical based on the fitting of experimental data on whole blood to the proposed correlations. There is considerable freedom observed in the choice of the proposed correlations in the literature [69], [46], [35]. The physical mechanisms behind these correlations and their relative validities are to be further investigated.

Fourthly, phase interactions are oversimplified indeed in the current contribution. As described above, interactions between various components in the blood are extremely complex, they exchange mass, momentum, and energy in a variety of ways with each other. Besides, the blood flow and vessel walls interact with each other considerably. When physiological factors are taken into account, these interactions are even more complicated.

Lastly, the physiological and medical implications of the computationally obtained results are to be noticed.

## 5 Conclusion

In this contribution, we are concerned with the computational modeling of blood flow through stenosed microvessels. A two-phase blood model is utilized with the continuous plasma phase described as a Newtonian fluid and the discrete RBC phase treated as a shear-thinning non-Newtonian fluid. Interactions between the plasma phase and the RBC phase are considered. The model is validated by the investigation of blood flow through microvessels with sudden expansion. After that, the model is applied to the blood flow through stenosed microvessels at different values of inlet hematocrits and velocity amplitudes. Volume fraction of the RBC phase in the vessel is obtained. A cell-free region on the downstream part of the stenosis is observed and local accumulation of the RBC phase is seen on the upstream part of the stenosis. With the increase inlet velocity amplitude *Vt*, it is found that the cell-rich layer downstream the stenosis exhibits an increase in length and thickness, while the recirculation zone near the downstream part of the stenosis expands largely in size. With the increase of the inlet hematocrit *Ht*, the size of the recirculation zone is not much affected, while the cell-rich layer downstream the stenosis increases in both length and thickness. As for wall shear stress, it is seen that its maximum value typically appears slightly before the maximum stenosis point of the microvessel. Also its maximum value scales up with increasing *Vt* and *Ht*. The obtained results validated the established computational model, and provide evidences in terms of the interaction between the RBC phase and the plasma phase. With the influence of flow conditions (*Vt*) and blood components *Ht* upon the wall shear stresses along vessel walls, helpful recommendations can be made for clinical applications.

## CRediT authorship contribution statement

**Yuhong Zhao:** Writing – original draft, Validation, Software, Investigation, Conceptualization. **Jue Xie:** Writing – review & editing, Project administration, Methodology, Investigation.

## Declaration of Competing Interest

The authors declare that they have no known competing financial interests or personal relationships that could have appeared to influence the work reported in this paper.
